# Editorial

**Published:** 1913-09

**Authors:** 


### EDITORIAL

The Business Office of the *Journal-Record* is 23 West Alabama Street.

The Editorial Office is 1014-15 Atlanta National Bank Building.

Address all Business Communications to *Journal-Record of Medicine,* 23 West Alabama Street.

Make remittances payable to *The Journal-Record of Medicine.*

On matters pertaining to the Editorial and Original Communications, address Edgar G. Ballenger, M. D., Atlanta, Ga.

Reprints of Original Articles will be furnished at cost price. Requests for the same should always be made in THE MANUSCRIPT.

We will present, postpaid, on request to each contributor of an original article, twenty (20) marked copies of *The Journal-Record of Medicine* containing such article.

### PROPHYLAXIS OF VULVO-VAGINITIS IN LITTLE GIRLS.

Disease approaches us through so many avenues and from so many directions that its prevention requires constant effort and careful searching observation. It is marvelous what we have done to protect ourselves, yet it is sometimes surprising what we have overlooked. Only prophetically clear vision will see in what is most plainly before us the things there that familiarity has made obscure, and show us the way to better conditions. It takes genius in a man to "see what he is looking at" anyhow, and the trained observers in these days of rapidly advancing medical science may well have genius ascribed to them.

We welcome, therefore, the suggestion made in an essay in this issue that the profession take up and most seriously investigate the condition of the nurses we employ for our infants. According to that writer specific vulvo-vaginitis makes horrid ravages among the children and institutes processes that endanger the health of these children so long as they live. It is also shown that treatment thus far is almost futile and that prevention alone can secure freedom from the disease.

The problem of the negro nurse for children is a live one, and its solution is fraught with inconvenience and danger. For generations she has been caring for the white children and many, many times has earned and held the affection of her charges long after they have passed from her care. But times are different and the present nurse is different. She has diseases that did not prevail a generation ago and specific, contagious vulvo-vaginitis is one of them. Moreover, she works in a different way and is farther from the supervision of the parents of her charges. This must be so or she could not do the things she is often guilty of. She has learned to quiet children by touching their genitalia and has been seen to do this on the public streets when a little one has fallen and hurt itself. If she has gonorrhoea, herself, it is practically certain that there are times when her fingers are infected with the coccus and that the disease may be conveyed in this manner. Thus, not only is there physical disease communicated directly to the infant, but the foundation for sexual hyperasthesias is laid and their attendant results of sexual perversions and immorality are foreordained.

Prophylaxis has never wanted for vigorous advocates when the way has been shown. The struggles in its behalf are among the greatest honors the Medical Profession has earned. Here is a new matter for earnest thought and constant labor. Educate the parents in the hygienic care the nurse should give the child and soon the demand will formulate the legislation Dr. Adkins suggests. There is a great—we will even say there is an awful need of clean and sanitary nurses for our children.

---

### NEW MEDICAL PRACTICE BOARD.

Governor Slaton has announced the appoinment of the new State Medical Board, as authorized by the Legislature at its summer session.

The allopathic appointments are all reappointments, but the homeopathic and eclectic members are new.

The two homeopaths are from Atlanta, for the reason that the other physicians of that school throughout the State united in a unanimous petition to the Governor that the Atlanta men be named.

The new board, through which the medical practice act will be administered, is as follows:

For Terms of One Year—Dr. J. W. Palmer, of Ailey, representing the regular school; Dr. C. M. Paine, of Atlanta, representing the homeopathic school.

For Terms of Two Years—Dr. F. M. Ridley, of LaGrange, representing the regular school; Dr. A. F. White, of Flovilla, representing the electic school; Dr. R. E. Hinman, of Atlanta, representing the homeopathic school.

For Terms of Three Years—Dr. N. Peterson, of Tifton, representing the regular school; Dr. O. B. Walker, of Bowman, representing the electic school.

For terms of Four Years—Dr. C. L. Nolan, of Marietta, representing the regular school; Dr. F. D. Patterson, of Cuthbert, representing the regular school; Dr. A. Fleming, of Waycross, representing the eclectic school.

We would suggest that the board use its influence and authority in stopping all infringements of the medical practice law at once so as to create the necessary respect for this law. A rigid enforcement from the beginning will mean a much easier time in the future. We have both the law and public sentiment with us.

### NOGUCHI'S DISCOVERY OF THE GERM OF HYDROPHOBIA.

In the current Journal of Experimental Medicine, Noguchi announces that he has found and reproduced the germ of hydrophobia.

Ever since Pasteur discovered the virus and began making injections for the prpevention of the disease, constant efforts have been made in the search for the germ itself in laboratories all over the world. This search is now ended and the task of securing a cure for the disease may be said to be well under way. It would appear that the germ is protozoon rather than a bacterial, and this of itself will at once change all ideas as to treatment. Cases of cure of the disease by the use of quinine have been reported, but not generally credited. It is possible that they may have been genuine if the disease is of protozoian type.

This discovery will open the way to greater activity in the search for the germs of measles, smallpox, scarlet fever, etc. We can in some measure protect ourselves from smallpox, but from the others we have no protection except running away, and no treatment but the symptomatic.

Noguchi's discovery adds another wreath to the laurels he already modestly wears, and were he to do nothing more, his work with syphilis and hydrophobia entitles him to undying fame in science, and endless graditude in the public mind. But he is a young man, and in all the years to come, we wish him continued opportunity and strength to use his skill and wisdom in the work he is doing.

Noguchi was born in Japan in 1876, and educated there; taking his degree in medicine at the Tokio Medical College in 1897. He took post graduate courses in this country and in Denmark, and returned to Japan to do special work with infectious diseases there. In 1909, he was appointed to the Rockefeller Institute for Experimental Research in this country, and has been at work in that capacity since then.

Sept. 11th, 1913.

EDITOR JOURNAL-RECORD OF MEDICINE:

We are calling upon the medical men of the South to help us make a collection of human embryos. Through the kindness of several men, in various parts of the country, a number of valuable specimens, ranging in age from three to seven weeks, are already at hand. The occasional ovum that you obtain in your private practice is the only source from which a collection large enough to be of any scientific service can be obtained.

The technical facilities we now have at our command for photographing, dissecting, sectioning and reconstructing embryological materials are such as will enable al lconcerned to derive the full benefit from the spepcimens. It is hoped that a careful study of the embryos will furnish information on the diseases of the ovum and uterus, as well as information on the development of the human embryo. Abortions and extrauterine pregnancies relieved by operation are the chief sources of normal and pathological ova.

The early stages are especially desirable, but any specimens of less than three months incubation or less than 3½ inches from vertex to breech will be appreciated.

The specimens should be placed in a wide mouth bottle containing a ten per cent. solution of formaldehyde. The ovum should be handled carefully, and should not be crowded or forced when it is placed in the bottle. The membranes should not be cut. The bottle should then be completely filled with the solution so as to exclude all air, securely corked, packed and sent by express c. o. d. All essential clinical data should be appended.

We will be pleased to furnish description notes of the embryos, information on special features, and in some cases photographs.

Address all materials and correspondence to:

DRS. J. L. CAMPBELL AND J. W. PAPEZ,
Atlanta Medical College,
Cor. Armstrong & Butler Sts., Atlanta, Ga.

